# Two New Terpenes Isolated from *Dictyostelium* Cellular Slime Molds

**DOI:** 10.3390/molecules25122895

**Published:** 2020-06-23

**Authors:** Hitomi Sasaki, Yuzuru Kubohara, Hirotaka Ishigaki, Katsunori Takahashi, Hiromi Eguchi, Akihiro Sugawara, Yoshiteru Oshima, Haruhisa Kikuchi

**Affiliations:** 1 Graduate School of Pharmaceutical Sciences, Tohoku University, 6-3, Aza-Aoba, Aramaki, Aoba-ku, Sendai 980-8578, Japan; sasaki.hitomi@toaeiyo.co.jp (H.S.); hiromi.e@me.com (H.E.); sugawara@mail.pharm.tohoku.ac.jp (A.S.); oshima@mail.pharm.tohoku.ac.jp (Y.O.); 2 Graduate School of Health and Sports Science, Juntendo University, 1-1 Hiraga-gakuendai, Inzai, Chiba 270-1695, Japan; ykuboha@juntendo.ac.jp; 3 Department of Medical Technology, Faculty of Health Science, Gunma Paz University, Takasaki 370-0006, Japan; ishigaki@paz.ac.jp (H.I.); k-takahashi@paz.ac.jp (K.T.)

**Keywords:** cellular slime molds, dictyostelid, natural products, terpenoids

## Abstract

We report a protoilludane-type sesquiterpene, mucoroidiol, and a geranylated bicyclogermacranol, firmibasiol, isolated from *Dictyostelium* cellular slime molds. The methanol extracts of the fruiting bodies of cellular slime molds were separated by chromatographic methods to give these compounds. Their structures have been established by several spectral means. Mucoroidiol and firmibasiol are the first examples of more modified and oxidized terpenoids isolated from cellular slime molds. Mucoroidiol showed moderate osteoclast-differentiation inhibitory activity despite demonstrating very weak cell-proliferation inhibitory activity. Therefore, cellular slime molds produce considerably diverse secondary metabolites, and they are promising sources of new natural product chemistry.

## 1. Introduction

Natural products, particularly those derived from microorganisms such as fungi and bacteria, have long played an essential role in the development of novel drugs [1] However, pharmaceutical research into natural products has recently declined because of factors such as increased difficulty in identifying new compounds with skeletally novel structures [2,3]. Thus, novel natural resources for natural products are required.

Cellular slime molds are a group of soil microorganisms that belong to the eukaryotic kingdom Amoebozoa, which is taxonomically distinct from fungi [4,5] The cellular slime mold *Dictyostelium discoideum* has been used as a model organism for studying eukaryotic cell functions because of its simple developmental pattern and ease of handling [6,7,8,9] Vegetative cells of *D. discoideum* grow as single ameba by eating bacteria. When these cells are starved, they initiate a developmental program of morphogenesis, forming a slug-shaped multicellular aggregate. This aggregate differentiates into two cell types, prespore and prestalk cells, which are precursors to spores and stalk cells, respectively. At the end of its development, the aggregate forms a fruiting body consisting of spores and a multicellular stalk [10].

We have focused on the utility of cellular slime molds as a source of natural compound [11] and have isolated α-pyronoids [12,13,14] amino sugar derivatives [15], and aromatics [16,17,18,19] with unique structures and various biological activities. For example, brefelamide [16] and its derivatives exhibit inhibitory effects on osteopontin expression [20,21] and immune checkpoint PD-L1 expression [22]. The above results indicate that cellular slime molds are an important source of lead compounds for drug discovery.

**Figure 1:**
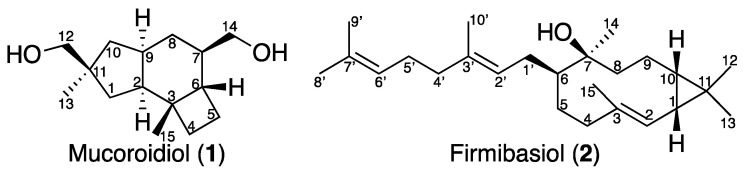
Structures of mucoroidiol (**1**) and firmibasiol (**2**).

In this paper, we report upon the isolation and structural elucidation of mucoroidiol (**1**), a protoilludane-type sesquiterpene from *D. mucoroides*, and firmibasiol (**2**), a geranylated sesquiterpene from *D. firmibasis* (Figure 1).

## 2. Results

### 2.1. Isolation and Structural Elucidation of Mucoroidiol

Multicellular fruiting bodies (80 g dry weight) of *D. mucoroides* Dm7 were cultured on agar plates in the presence of 0.5 mM ZnCl_2_ [23]. They were extracted three times with methanol at room temperature to yield an extract (10 g), which was then partitioned between ethyl acetate and water. The fraction soluble in ethyl acetate (2.8 g) was separated by silica-gel column chromatography and gel permeation column chromatography to afford mucoroidiol (**1**) (1.3 mg).

**Figure 2:**
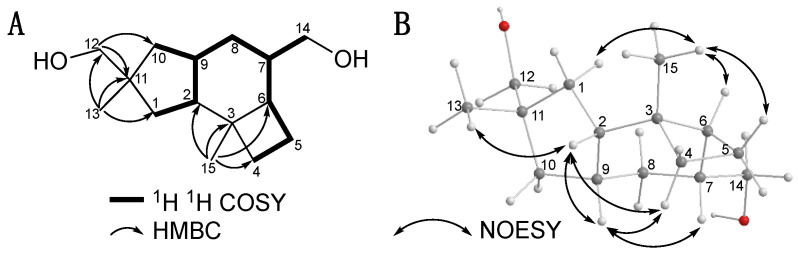
The structural elucidation of mucoroidiol (**1**). (**A**). Planar structure of **1** and representative correlations of ^1^H-^1^H COSY and HMBC spectra. (**B**). Relative structure of **1** and representative correlations of NOESY spectrum.

**Scheme 1:**
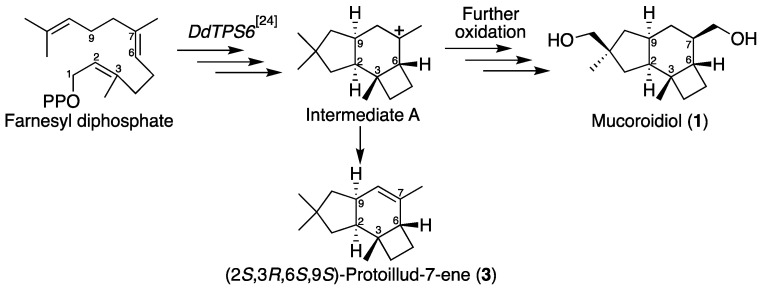
The plausible biosynthetic pathway of mucoroidiol (**1**).

HRFAB-MS (*m*/*z* 239.2011 [M+H]^+^) indicated the molecular formula of **1** as C_15_H_26_O_2_. The NMR spectra of **1** are shown in Supplementary Materials (Pages S2~S4). The ^13^C NMR spectrum of **1** showed the presence of two quaternary, four methine, seven methylene, and two methyl carbons (Table 1). The ^1^H-^1^H COSY correlations revealed the connectivity of C-1–C-2–C-9(–C-10)–C-8–C-7(–C-14)–C-6–C-5–C-4. The HMBC correlations of H_3_-15 to C-2, C-3, C-4 and C-6; H_2_-12 to C-1, C-10 and C-11; and H_3_-13 to C-1, C-10 and C-11 confirmed the protoilludane skeleton of **1** (Figure 2A). The cross-peaks of H-2 to H-4α, H-9, and H_3_-13, as well as those of H-9 to H-4α and H-7, revealed that these protons faced the α-plane, and that the relative configurations at C-2, C-7, C-9 and C-11 are determined as S*, R*, R* and R*, respectively. Conversely, correlations from H_3_-15 to H-1β and H-5β in the NOESY spectrum revealed that these protons faced the β-plane, and that the relative configurations at C-3 and C-6 are determined as R* and S*, respectively (Figure 2B). The yield of **1** was so small that its absolute configuration could not be determined by chemical conversion. On the other hand, it was reported that the incubation of DdTPS6 (a terpene cyclase of *D. discoideum*) with farnesyl diphosphate afforded (2*S*,3*R*,6*S*,7*R*,9*S*)-protoillud-7-ene (**3**) via carbocation intermediate A (Scheme 1) [24]. Some terpene cyclases of *D. mucoroides* would be evolutionary common with those of *D. discoideum*, indicating that the absolute configurations of **1** should be common with **3**. Thus, the absolute configurations of **1** are assumed to be 2*S*, 3*R*, 6*S*, 7*R*, 9*R,* and 11*R*.

### 2.2. Isolation and Structural Elucidation of Firmibasiol

Multicellular fruiting bodies (48 g dry weight) of the cellular slime mold (*D. firmibasis* 91HO-33) were cultured on plates and extracted three times with methanol at room temperature to yield an extract (11 g), which was partitioned between ethyl acetate and water. The fraction soluble in ethyl acetate (2.3 g) was separated by repeated column chromatography over silica gel and octadecyl silica gel to yield firmibasiol (**2**) (1.8 mg).

**Figure 3:**
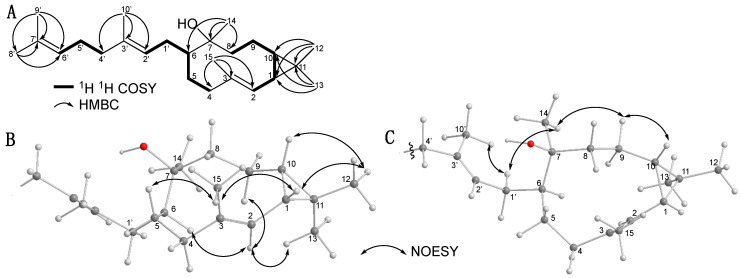
The structural elucidation of firmibasiol (**2**) (**A**). Planar structure of **2** and representative correlations of ^1^H-^1^H COSY and HMBC spectra. (**B**,**C**). Relative structure (side view (**B**) and top view (**C**)) of **2** and representative correlations of the NOESY spectrum.

**Scheme 2:**
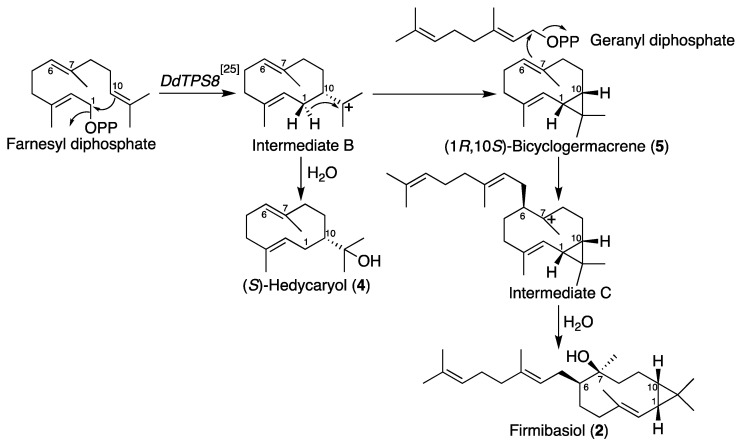
The plausible biosynthetic pathway of firmibasiol (**2**).

HREI-MS (*m*/*z* 358.3258 [M]^+^) indicated the molecular formula of **2** as C_25_H_42_O. The NMR spectra of **2** are shown in Supplementary Materials (Pages S5~S7). The ^13^C NMR spectrum of **2** showed the presence of six olefinic, two quaternary, three methine, seven methylene, and seven methyl carbons (Table 2). The HMBC correlations of H_3_-15 to C-2, C-3 and C-4; H_3_-14 to C-6, C-7 and C-8; H_3_-12 to C-1, C-10 and C-11; and H_3_-13 to C-1, C-10 and C-11 connect the partial structures confirmed by the ^1^H-^1^H COSY spectrum established the bicyclogermacrane moiety of **2** (Figure 3A). In addition, the HMBC correlations of H_3_-10′ to C-2′, C-3′ and C-4′; and H_3_-9′ to C-6′, C-7′ and C-8′revealed the 1-geranylated bicyclogermacrane structure of **2**. The cross-peaks of H_3_-12–H-10, H_3_-12–H-1, H-1–H_3_-15, and H_3_-15–H-5β in the NOESY spectrum revealed that these protons faced the β-plane (Figure 3B). Since H-1, H-10, and H_3_-12 direct to the same side, the relative configurations at C-1 and C-10 are determined as R* and S*, respectively. Conversely, the cross-peaks of H_3_-13–H-2, H-2–H-6, and H-2–H-9α indicated that these protons faced the α-plane, determining that the olefin between C-2 and C-3 has an *E*-configuration, and the relative configuration at C-6 is S*. In addition, the cross-peaks of H-9β–H_3_-14 and H_3_-14–H_2_-1′ indicated that these protons faced the same direction (Figure 3C), indicating that the relative configuration at C-7 is S*. In addition, the NOESY cross-peak between H-1′ and H_3_-10′ revealed that the olefin between C-2′ and C-3′ also has an *E*-configuration. The yield of **2** was very small such that its absolute configuration could not be determined by chemical conversion. On the other hand, it was reported that the incubation of DdTPS8 (a terpene cyclase of *D. discoideum*) with farnesyl diphosphate afforded (*S*)-hedycaryol (**4**) via carbocation intermediate B (Scheme 2) [25]. Firmibasiol (**2**) is presumed to be biosynthesized from intermediate B, which would be converted into (1*R*,10*S*)-bicyclogermacrene (**5**). Subsequently, the C-6–C-7 olefin attacks geranyl diphosphate, and water addition to the carbocation intermediate C at C-7 produces **2**. Through this plausible biosynthetic route, the absolute configurations of C-10 in **2** should be retained from carbocation intermediate B. Therefore, the absolute configurations of **2** are assumed to be 1*R*, 6*S*, 7*S*, and 10*S*. Although firmibasiol (**2**) contains five isoprene units (C_25_), it is not a sesterterpene, but rather a geranylated sesquiterpene. Prenylated terpenoids such as **2** are very rare types of natural compounds; only one compound has been reported so far [26].

### 2.3. Biological Activity of Compounds 1 and 2

**Figure 4:**
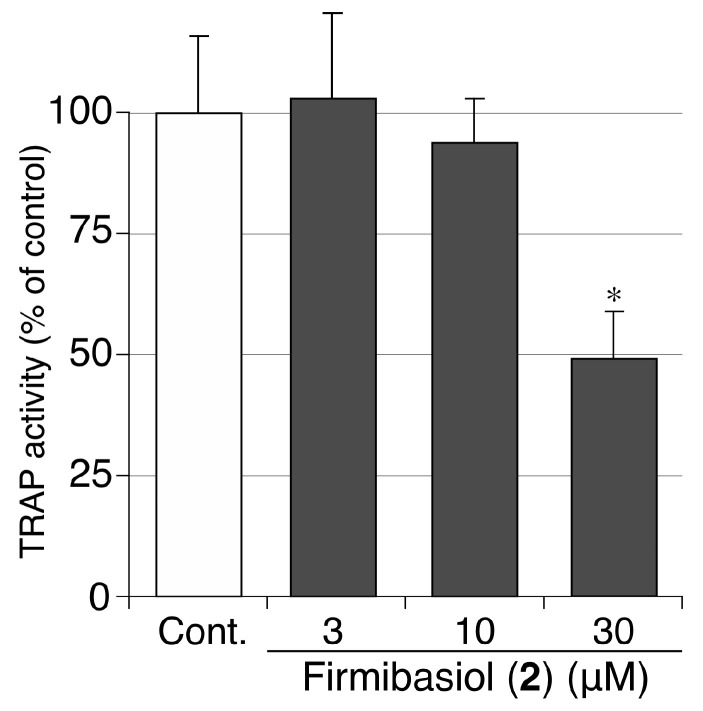
Osteoclastogenesis-suppressive activity of **2**.

Mucoroidiol (**1**) and firmibasiol (**2**) were screened to investigate several types of biological activities. Osteoclasts are multinucleated cells that resorb bone tissue. They are formed by the fusion of mononuclear monocyte/macrophage lineage precursor cells. Excessive bone resorption often results in osteoporosis and rheumatoid arthritis [27]. Firmibasiol (**2**) showed moderate receptor activator of NF-κB ligand (RANKL)-induced osteoclast-differentiation inhibitory activity (IC_50_ 28 μM) by measurement of activity of tartrate-resistant acid phosphatase (TRAP) [28] (Figure 4), while mucoroidiol (**1**) did not show remarkable inhibitory activity. On the other hand, mucoroidiol (**1**) and firmibasiol (**2**) exhibited weak anti-proliferative activity against HeLa cells (IC_50_ > 40 μM), but did not show apparent anti-bacterial activities both in Gram-positive (*Staphylococcus aureus*) and Gram-negative (*Escherichia coli*) bacteria (Table 3).

The data are expressed as percentages in relation to the mean value of the control cells. The bars indicate the standard deviation of the three wells. The statistical significance of the differences was determined by Welch’s *t*-test. * *p* < 0.05 vs. control.

## 3. Discussion

Recently, a phylogenetic analysis revealed that terpene cyclase genes exist in several species of cellular slime molds [29]. *D. discoideum* emits volatiles containing several types of terpenoid hydrocarbons. These hydrocarbons were produced by the incubation of the recombinant terpene cyclase with farnesyl or geranylgeranyl diphosphate [24,25,30]. However, unlike when only terpene cyclase acted, the multicellular fruiting bodies of a cellular slime mold should synthesize several types of modified terpenes converted by various biosynthetic enzymes in vivo. Mucoroidiol (**1**) is the first example of a terpene diol obtained from cellular slime molds. Firmibasiol (**2**) is a type of prenylated terpenoid, which is a very rare type of natural compound. Because the isolated amounts of compounds **1** and **2** were very small, their absolute configuration could not be determined. Instead, they are assumed by biosynthetic similarity with terpenes obtained by terpene cyclases of *D. discoideum*. The determination of their absolute configuration should be made by de novo synthetic studies in the future. On the other hand, firmibasiol (**2**) showed moderate osteoclast-differentiation inhibitory activity, and can be used as a seed compound for anti-osteoporosis drugs. Therefore, these cellular slime molds are promising sources of new natural product chemistry.

## 4. Materials and Methods

### 4.1. General Methods

Analytical TLC was performed on silica gel 60 F_254_ (Merck). Silica gel column chromatography was carried out on silica gel 60 (70–230 mesh, Merck). Octadecyl silica gel column chromatography was carried out on Cosmosil 140C_18_-OPN (NACALAI TESQUE, Inc., Kyoto Japan). NMR spectra were recorded on JEOL ECA-600. Chemical shifts for ^1^H and ^13^C NMR are given in parts per million (δ) relative to tetramethylsilane (δH 0.00) and residual solvent signals (δC 77.0) as internal standards. Mass spectra were measured on JEOL JMS-700 and JMS-DX303.

### 4.2. Organism and Culture Conditions

*Dictyostelium mucoroides* Dm7 was provided by NBRP Nenkin (https://nenkin.nbrp.jp/). Its spores were cultured at 22 °C with *Klebsiella aerogenes* on A-medium consisting of 0.5% glucose, 0.5% polypeptone, 0.05% yeast extract, 0.225% KH2PO4, 0.137% Na2HPO4 × 12H2O, 0.05% MgSO4 × 7H2O, and 1.5% agar. *Dictyostelium firmibasis* 91HO–33 was kindly supplied by Dr. Hiromitsu Hagiwara, National Science Museum, Tokyo, Japan, and has been deposited into NBRP Nenkin. Its spores were cultured at 22 °C with *Escherichia coli* on A-medium. When fruiting bodies had formed after four days, they were harvested for extraction.

### 4.3. Isolation of Mucoroidiol (**1**)

The fruiting bodies (dry weight 80 g) of *D. mucoroides* Dm7 were collected after cultured in A-medium with 0.5 mM Zinc (II) chloride. They were extracted three times with methanol at room temperature to give an extract (10 g), which was then partitioned between ethyl acetate and water to yield ethyl acetate solubles (2.8 g). The ethyl acetate solubles were chromatographed over silica gel, and the column was eluted with hexane–ethyl acetate mixtures with increasing polarity to afford hexane-ethyl acetate (1:3) eluent (fraction A, 67 mg). Fraction A was further separated by octadecyl silica gel column using water–acetonitrile solvent system to give water–acetonitrile (1:1) elutant (fraction B, 28 mg). Fraction B was subjected to recycle preparative HPLC (column, GPC-T-2000 (φ 20 mm × 600 mm, YMC Co., Ltd.); solvent, ethyl acetate) to give mucoroidiol (**1**) (1.3 mg). Data for **1**: colorless amorphous solid; [α]_D_^24^ -53.4 (c 0.13, chloroform); ^1^H NMR and ^13^C NMR spectroscopic data are shown in Table 1; HRFABMS *m/z* 239.2011 [M + H]^+^ (239.2010 calculated for C_15_H_27_O_2_).

### 4.4. Isolation of Firmibasiol (**2**)

The fruiting bodies (dry weight 48 g) of *D. firmibasis* 91HO-33 were collected after cultured in A-medium. They were extracted three times with methanol at room temperature to give an extract (11 g), which was then partitioned with ethyl acetate and water to yield ethyl acetate solubles (2.3 g). The ethyl acetate solubles were chromatographed over silica gel and the column was eluted with hexane–ethyl acetate mixtures of increasing polarity to afford hexane-EtOAc (17:3) eluent (fraction C, 356 mg). Fraction C was separated by ODS column using water–acetonitrile solvent system to give water–acetonitrile (1:4) elutant (fraction D, 20 mg). Fraction D was further separated by silica gel column using chloroform to give firmibasiol (**2**) (1.8 mg). Data for **2**: colorless amorphous solid; [α]_D_^26^ -30.9 (c 0.16, chloroform); ^1^H NMR and ^13^C NMR spectroscopic data are shown in Table 2; HREIMS *m/z* 358.3258 [M]^+^ (358.3236 calculated for C_25_H_42_O).

### 4.5. Cell Proliferation Assay

Human cervical cancer HeLa cells were grown and maintained at 37 °C (5% CO_2_ in air) in Dulbecco’s modified Eagle’s medium (DMEM) (Catalog No. D5796, Sigma-Aldrich) supplemented with 10% (*v*/*v*) fetal bovine serum (FBS). For cell proliferation assay, HeLa cells were incubated for 3 days in 12 well plates (5 × 10^3^ cells/well), with each well containing 1 mL of DMEM (10% FBS) and the additives in duplicate; the additives were 0.2% (*v*/*v*) dimethyl sulfoxide (DMSO), 20–40 μM of mucoroidiol (**1**) or firmibasiol (**2**). The relative cell number was assessed using Alamar blue (cell number indicator) and half maximal (50%) inhibitory concentration (IC_50_) of **1** and **2** was determined as described previously [31].

### 4.6. Measurement of Minimum Inhibitory Concentration (MIC)

The Gram-positive bacteria methicillin-susceptible *Staphylococcus aureus* (MSSA; strain ATCC29213), methicillin-resistant *S. aureus* (MRSA; ATCC43300) and the Gram-negative bacterium *Escherichia coli* (ATCC25922) were used in this study. The bacteria suspended in Mueller-Hinton broth (5 × 10^5^ CFU/mL; 0.1 mL/well) were incubated for 24 h at 37 °C in 96-well plates (Corning, NY, USA) in the presence of vehicle, various concentrations of serially diluted test compounds, or known antibiotics; MIC was defined as the lowest concentration of the additives that inhibited visible bacterial growth.

### 4.7. Osteoclast Differentiation Experiments

RAW264 cells were grown in α-MEM containing 10% fetal bovine serum and passaged every 3 days. To differentiate into osteoclast, RAW264 cells were seeded at 4 × 10^3^ cells/well in 96-well plates and cultured in the presence of RANKL (50 ng/mL) and each compound for 4 days. The cells were sequentially fixed with 10% formalin for 10 min and ethanol for 1 min, and then dried. To measure the activity of tartrate-resistant acid phosphatase (TRAP), which is a marker enzyme of osteoclastogenesis, fixed cells were incubated in 100 µL of citrate buffer (50 mM, pH 4.6) containing 10 mM tartrate and 5 mM *p*-nitrophenylphosphate for 30–60 min and the reaction mixtures were transferred into another well containing 100 µL of 0.1 M NaOH solution. The absorbances at 405 nm were measured as TRAP activity.

## Figures and Tables

**Table 1:** NMR spectral data of mucoroidiol (**1**) ^a^.

	^13^C	(DEPT)	^1^H
1	37.0	CH_2_	1.23 (1H, dd, *J* = 12.5, 6.5 Hz)
			1.32–1.37 (1H, m)
2	45.6	CH	1.84 (1H, dt, *J* = 12.9, 6.5 Hz)
3	39.0	C	
4	31.7	CH_2_	1.42–1.47 (1H, m)
			2.03 (1H, q, *J* = 9.4 Hz)
5	24.4	CH_2_	1.33–1.37 (1H, m)
			2.14–2.21 (1H, m)
6	40.2	CH	1.33–1.39 (1H, m)
7	44.7	CH	1.60–1.63 (1H, m)
8	30.3	CH_2_	0.78 (1H, q, *J* = 12.9 Hz)
			1.35–1.41 (1H, m)
9	38.4	CH	2.10–2.16 (1H, m)
10	42.8	CH_2_	1.30–1.35 (1H, m)
			1.59 (1H, dd, *J* = 13.5, 7.8 Hz)
11	41.7	C	
12	73.0	CH_2_	3.34 (1H, d, *J* = 10.6 Hz)
			3.37 (1H, d, *J* = 10.6 Hz)
13	26.5	CH_3_	0.99 (3H, s)
14	67.2	CH_2_	3.32–3.37 (1H, m)
			3.51 (1H, dd, *J* = 10.5, 4.8 Hz)
15	26.3	CH_3_	1.11 (3H, s)

^a^ 600 MHz for ^1^H and 150 MHz for ^13^C in CDCl_3_.

**Table 2:** NMR spectral data of firmibasiol (**2**) ^a^.

	^13^C	(DEPT)	^1^H
1	25.1	CH	1.28 (1H, t, *J* = 9.0 Hz)
2	121.0	CH	4.88 (1H, d, *J* = 9.0 Hz)
3	137.2	C	
4	40.5	CH_2_	1.80 (1H, t, *J* = 12.0 Hz)
			2.07–2.11 (1H, m)
5	26.7	CH_2_	1.18–1.25 (1H, m)
			1.44–1.51 (1H, m)
6	42.3	CH	1.41–1.45 (1H, m)
7	76.0	C	
8	38.3	CH_2_	1.18–1.27 (2H, m)
9	21.3	CH_2_	0.58 (1H, q, *J* = 12.0 Hz)
			1.50–1.54 (1H, m)
10	27.8	CH	0.66 (1H, ddd, *J* = 12.0, 9.0, 3.6 Hz)
11	20.1	C	
12	28.8	CH_3_	1.04 (3H, s)
13	15.1	CH_3_	1.11 (3H, s)
14	25.8	CH_3_	1.07 (3H, s)
15	17.3	CH_3_	1.73 (3H, s)
1′	30.2	CH_2_	2.08–2.14 (2H, m)
2′	123.1	CH	5.27 (1H, t, *J* = 7.2 Hz)
3′	136.2	C	
4′	40.0	CH_2_	2.03 (2H, t, *J* = 7.2 Hz)
5′	26.8	CH_2_	2.05–2.10 (2H, m)
6′	124.3	CH	5.01 (1H, tq, *J* = 7.2, 1.2 Hz)
7′	131.5	C	
8′	25.7	CH_3_	1.68 (3H, d, *J* = 7.2, 1.2 Hz)
9′	17.7	CH_3_	1.60 (3H, s)
10′	16.3	CH_3_	1.63 (3H, s)

^a^ 600 MHz for ^1^H and 150 MHz for ^13^C in CDCl_3._

**Table 3:** Antitumor and anti-bacterial activities of **1** and **2** ^a^.

	IC_50_ (μM) vs.		MIC (μM) vs.	
	HeLa	*S. aureus* (MSSA)	*S. aureus* (MRSA)	*E. coli*
Mucoroidiol (**1**)	>40	>100	>100	>100
Firmibasiol (**2**)	>40	>100	>100	>100

^a^ Half maximal (50%) inhibitory concentration (IC_50_) (versus HeLa cells) and minimum inhibitory concentration (MIC) (versus *S. aureus* and *E. coli*) of **1** and **2** were assessed as described in the Section 4.

## Supplementary Materials

The NMR spectra of new compounds **1** and **2** are available online at https://www.mdpi.com/1420-3049/25/12/2895/s1.
