# Supplementary material for: Two New Terpenes Isolated from Dictyostelium Cellular Slime Molds
Source: Molecules. 2020 Jun 23;25(12):2895. doi: 10.3390/molecules25122895 (PMC7356884; doi:10.3390/molecules25122895)
Supplement: Supplementary file 1 [file molecules-25-02895-s001.pdf]

Supplementary Materials

Novel Two Terpenes Isolated from *Dictyostelium* Cellular Sime Molds

Hitomi Sasaki,[1] Yuzuru Kubohara,[2] Hirotaka Ishigaki,[3] Katsunori Takahashi,[3] Hiromi Eguchi,[1] Akihiro Sugawara,[1] Yoshiteru Oshima[1] and Haruhisa Kikuchi[1,*]

[1] *Graduate School of Pharmaceutical Sciences, Tohoku University, 6-3, Aza-Aoba, Aramaki, Aoba-ku, Sendai 980-8578, Japan.*

[2] *Graduate School of Health and Sports Science, Juntendo University, 1-1 Hiraga-gakuendai, Inzai, Chiba 270-1695, Japan*

[3] *Department of Medical Technology, Faculty of Health Science, Gunma Paz College, Takasaki 370-0006, Japan*

* Correspondence: hal@mail.pharm.tohoku.ac.jp; Tel.: +81-22-795-6824

Table of Contents

S2 ~ S4 NMR spectra of mucoroidiol (**1**)

S5 ~ S7 NMR spectra of firmibasiol (**2**)

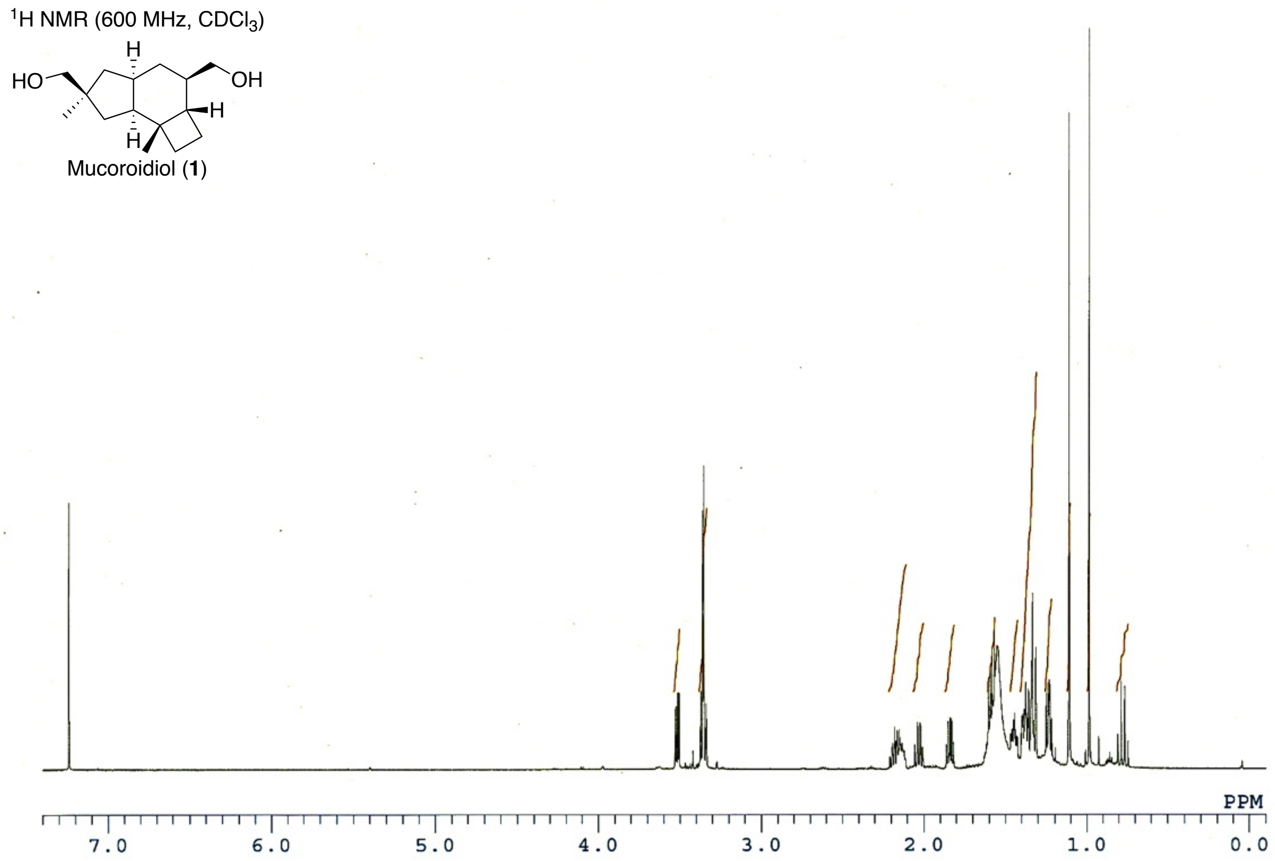

^{1}H NMR (600 MHz, $CDCl_3$)
HO
OH
H
Mucoroidiol (1)
PPM
7.0
6.0
5.0
4.0
3.0
2.0
1.0
0.0

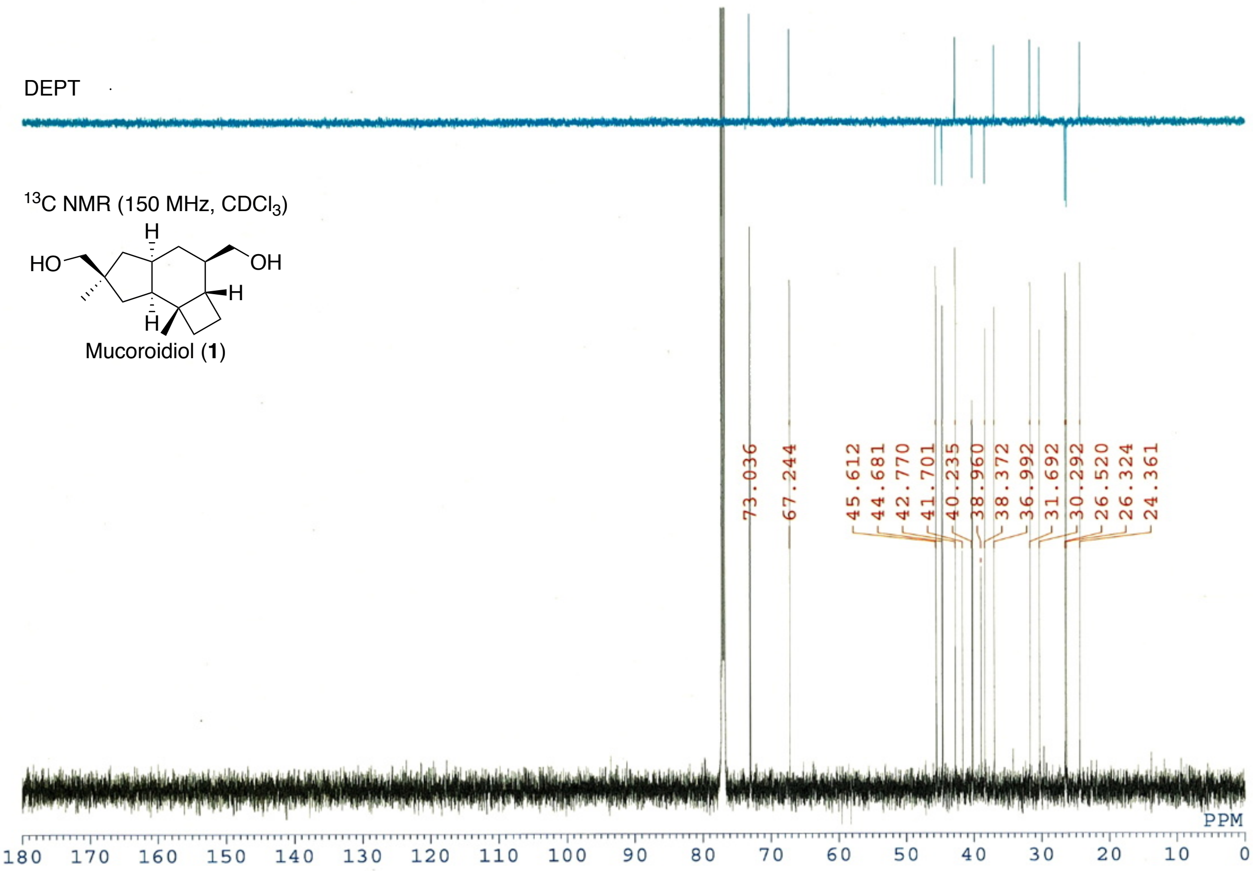

DEPT
^{13}C NMR (150 MHz, $CDCl_3$)
HO
OH
H
Mucoroidiol (1)
73.036
67.244
45.612
44.681
42.770
41.701
40.235
38.960
38.372
36.992
31.692
30.292
26.520
26.324
24.361
PPM
180
170
160
150
140
130
120
110
100
90
80
70
60
50
40
30
20
10
0

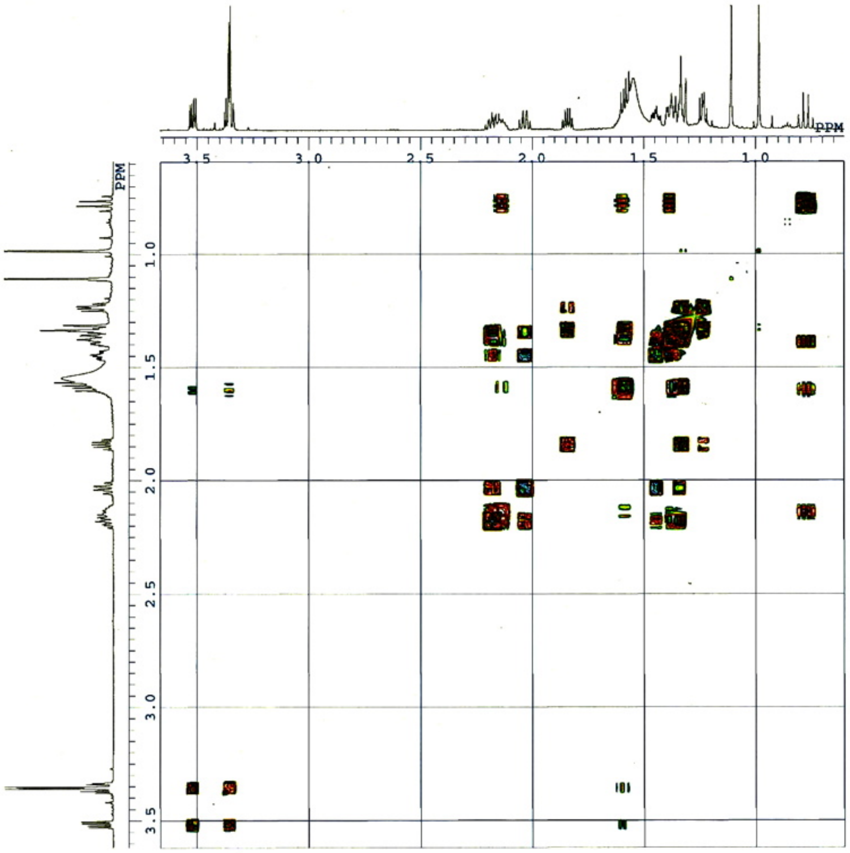

DQFCOSY

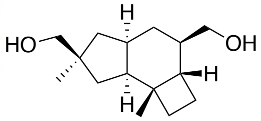

Mucoroidiol (**1**)

HMQC spectrum

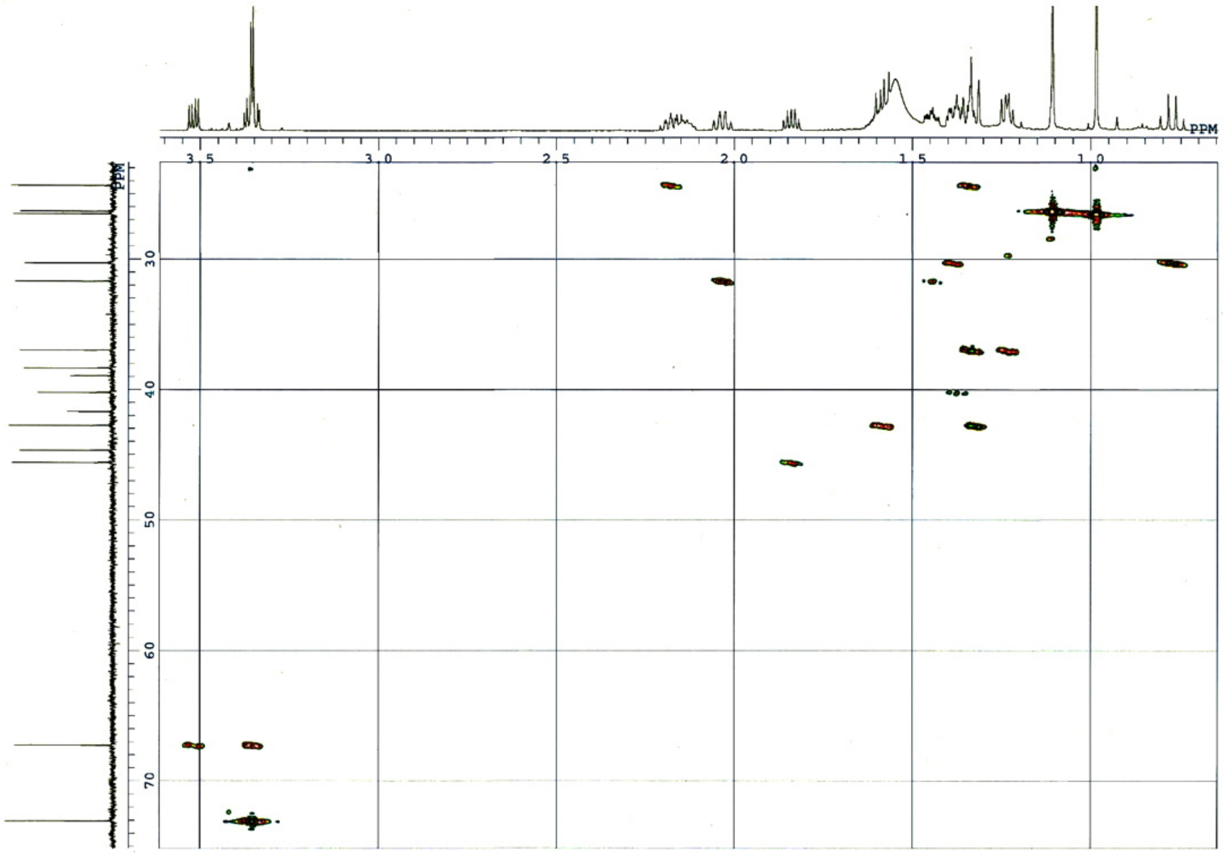

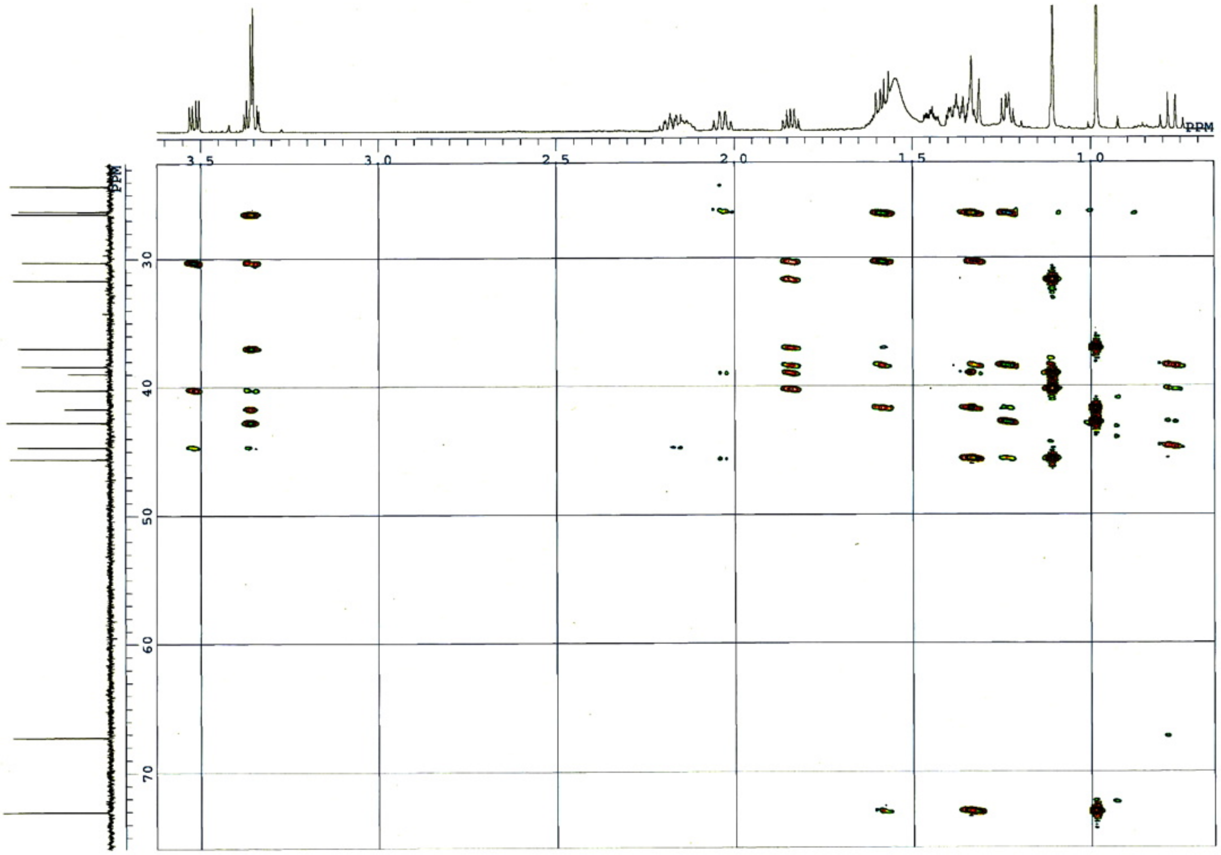

HMBC spectrum

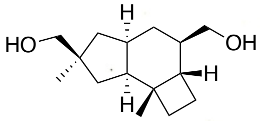

Mucoroidiol (**1**)

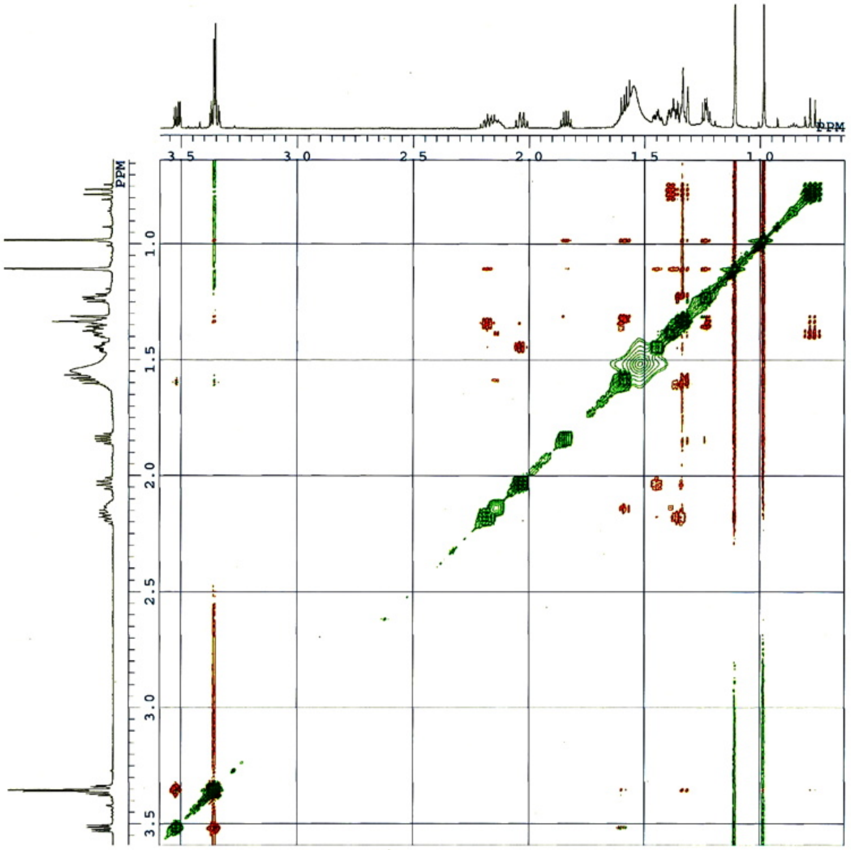

NOESY spectrum

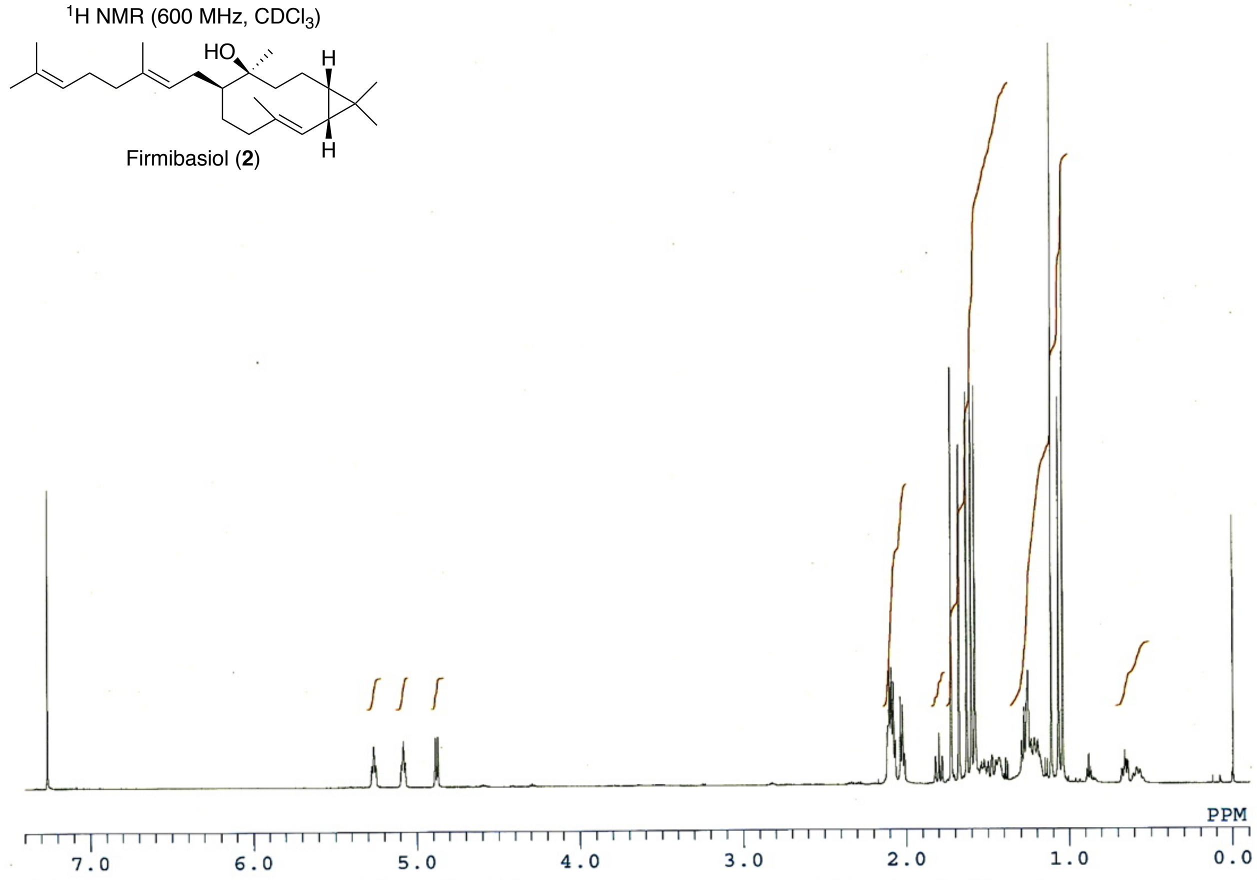
1H NMR (600 MHz, CDCl3)
HO
H
H
Firmibasiol (2)
PPM
7.0
6.0
5.0
4.0
3.0
2.0
1.0
0.0

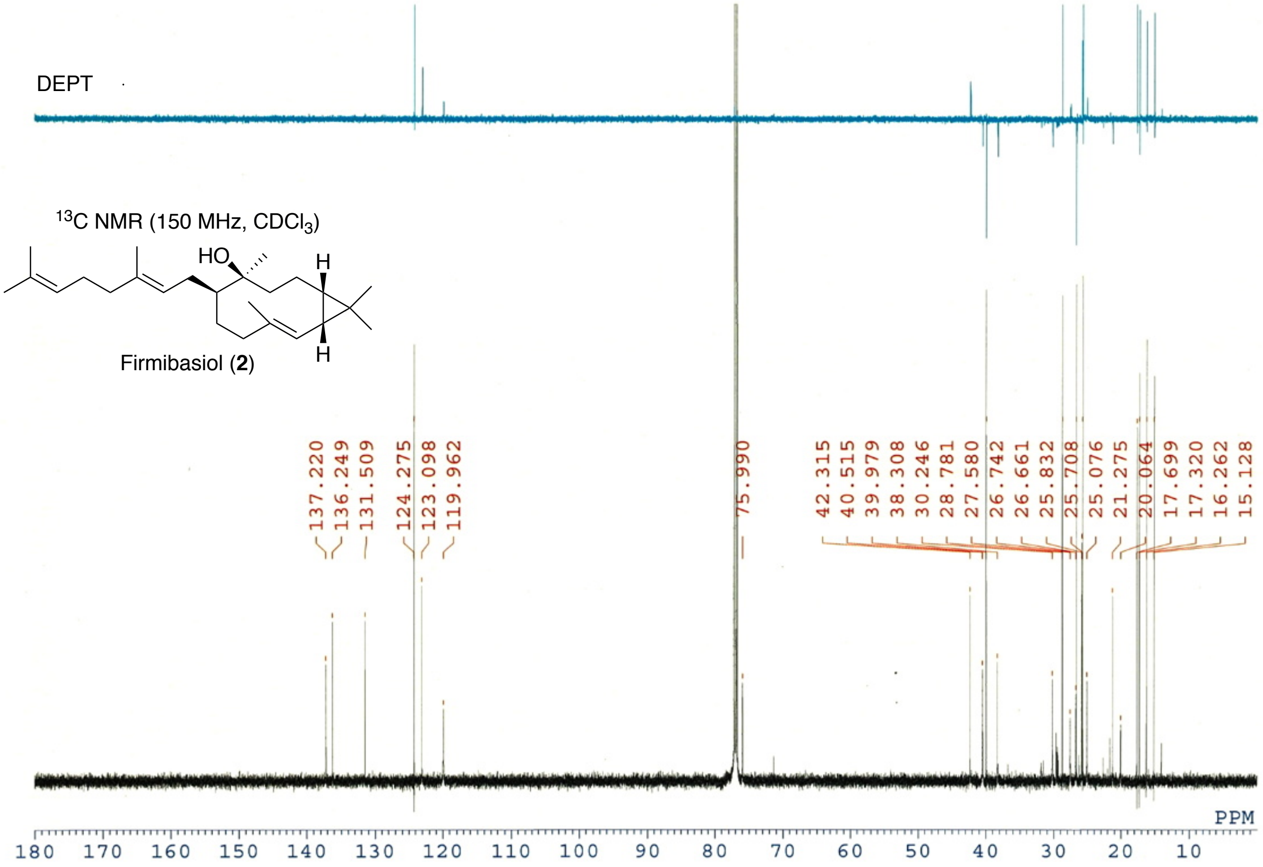
DEPT
13C NMR (150 MHz, CDCl3)
HO
H
H
Firmibasiol (2)
137.220
136.249
131.509
124.275
123.098
119.962
75.990
42.315
40.515
39.979
38.308
30.246
28.781
27.580
26.742
26.661
25.832
25.708
25.076
21.275
20.064
17.699
17.320
16.262
15.128
PPM
180
170
160
150
140
130
120
110
100
90
80
70
60
50
40
30
20
10

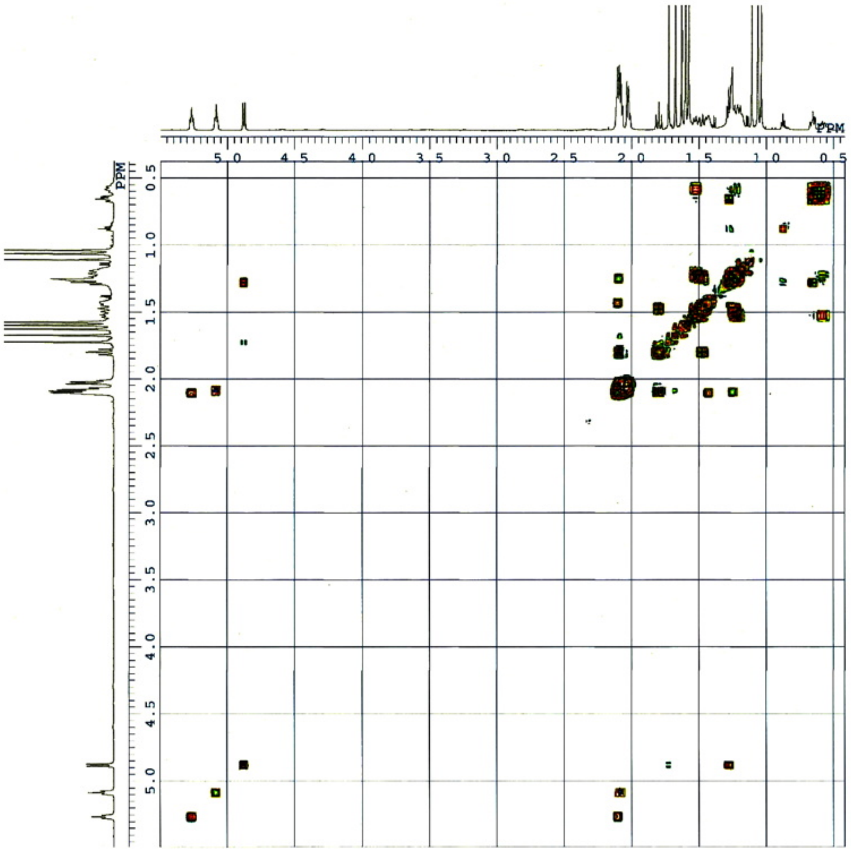

DQFCOSY

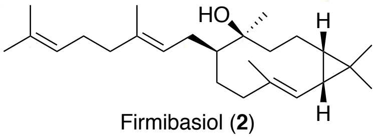

Firmibasiol (**2**)

HMQC spectrum

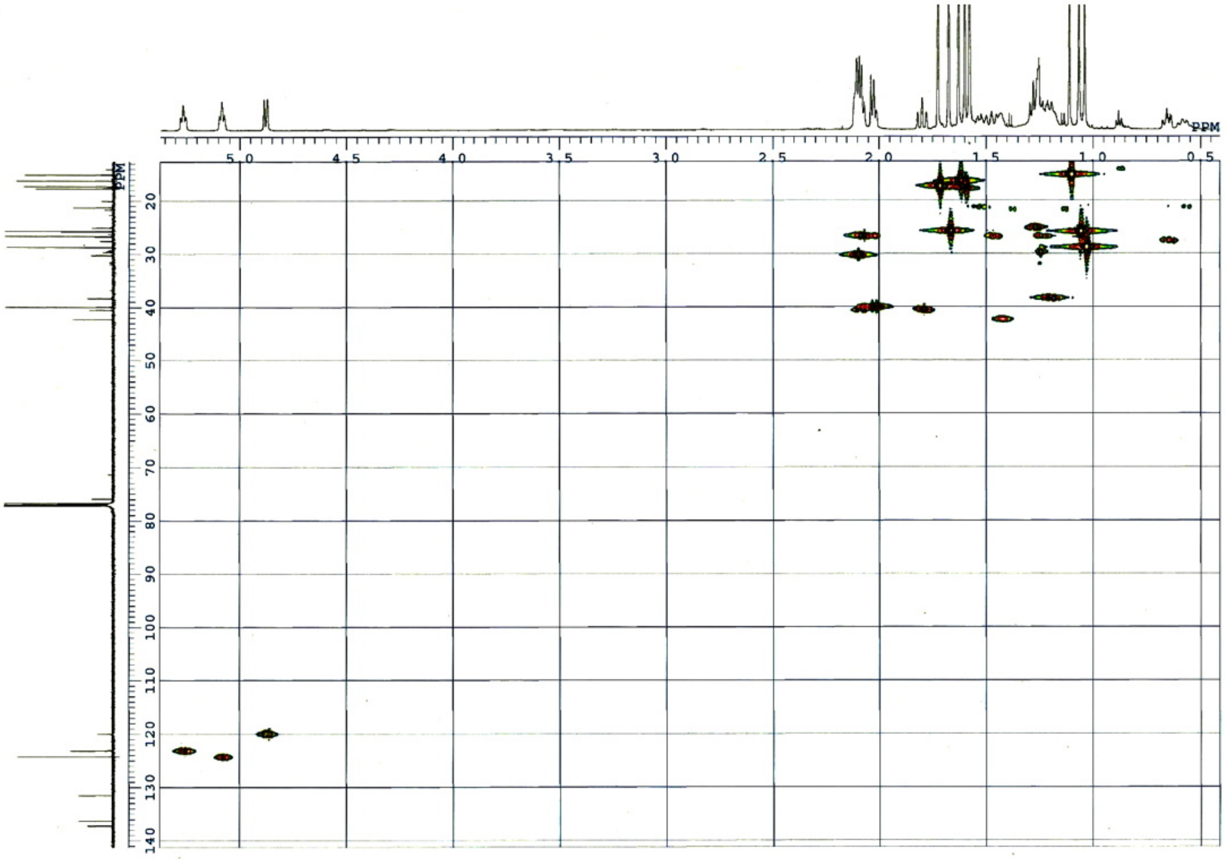

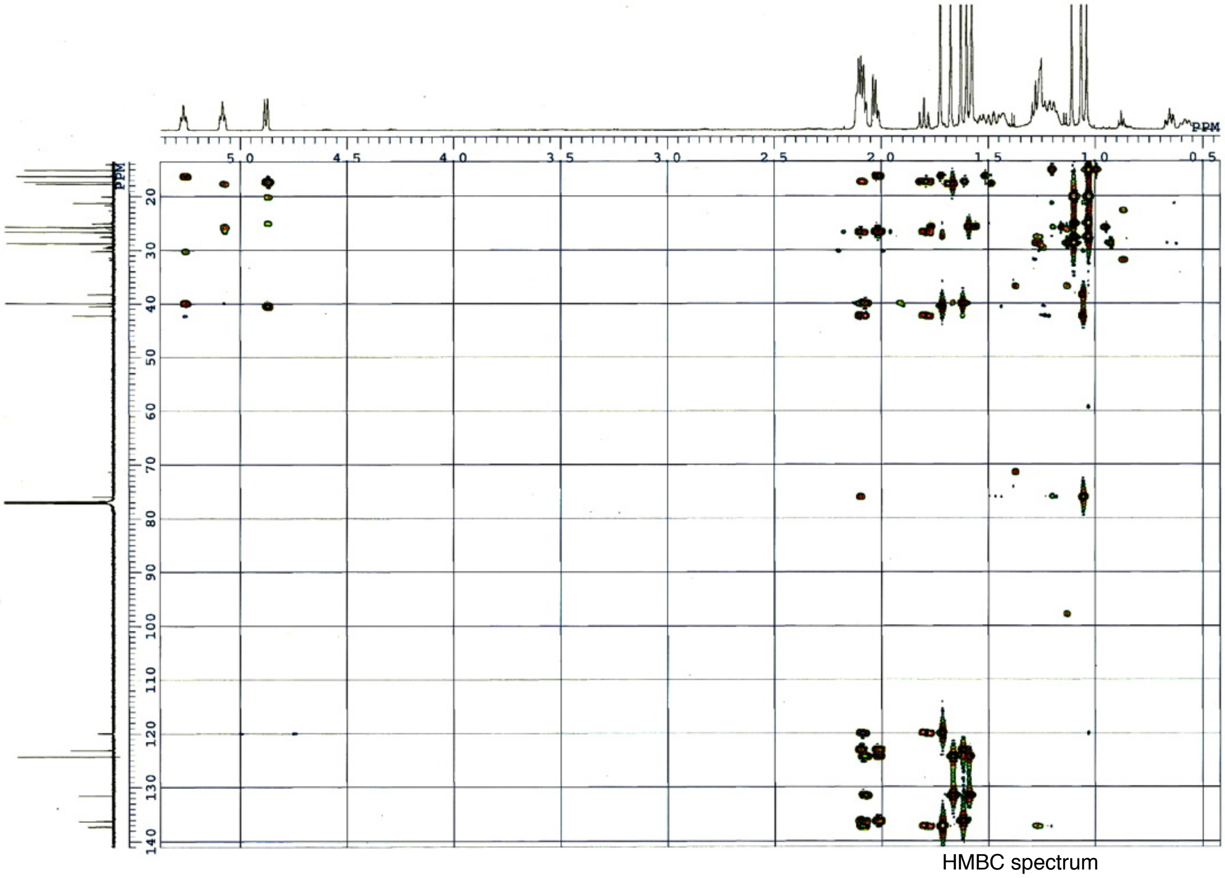

HMBC spectrum

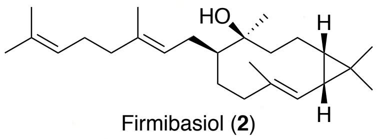

Firmibasiol (**2**)

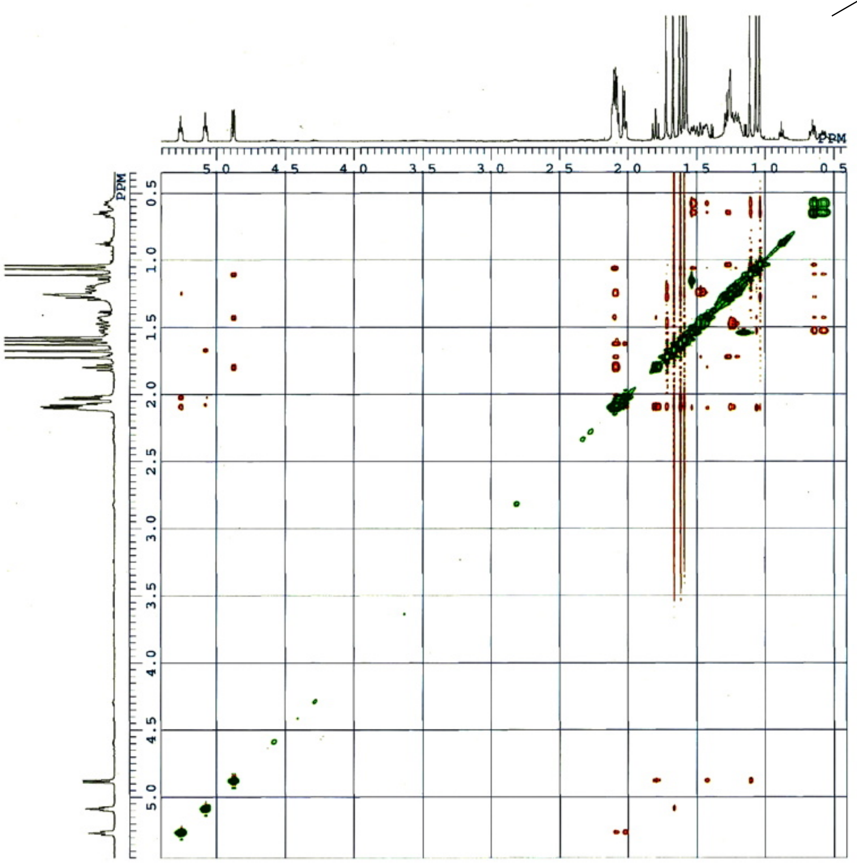

NOESY spectrum
